# Design and Evaluation of Ocular Controlled Delivery System for Diclofenac Sodium

**Published:** 2015

**Authors:** Zahra Jafariazar, Nasim Jamalinia, Fatemeh Ghorbani-Bidkorbeh, Seyed Alireza Mortazavi

**Affiliations:** a *Department of Pharmaceutics, Pharmaceutical Sciences Branch, Islamic Azad University, Tehran, Iran. *; b *Department of Pharmaceutics, School of Pharmacy, Shahid Beheshti University of Medical Sciences, Tehran, Iran.*

**Keywords:** Ocular film, Controlled release, Diclofenac sodium

## Abstract

Diclofenac sodium as ophthalmic dosage form is used for the treatment of the pain, swelling and redness of patients’ eyes recovering from cataract surgery; however, it faces the bioavailability limitation of eye drops due to effective protective mechanisms and corneal barrier functions in the eyes. Therefore, this investigation was aimed to develop ocular film formulations to achieve controlled drug release. Drug films were prepared using polymers, namely hydroxypropyl methylcellulose (HPMC) and polyvinyl pyrrolidone (PVP), Eudragit RL PO, and Eudragit RS PO by solvent casting method considering parameters such as drug: polymer ratio, different polymer combinations as well as plasticizer effect. Ocular films were evaluated for various physicochemical parameters such as physical characters, film thickness, uniformity of weight, drug content, swelling index, mucoadhesion time and *in-vitro* release study. Ocular films complied with all physicochemical parameters underwent *in-vitro* release study. Finally, the film formulation with HPMC: Eudragit RS PO 1:1 ratio, Drug: Polymer ratio 1:45 and glycerin as plasticizer showed controlled and prolonged release following the zero order and non-Fickian transport.

## Introduction

Due to the poor drug bioavailability, ocular drug delivery still faces many challenges which can be the result of ocular anatomy, physiology and efficient protective mechanism including extensive nasolachrymal drainage, tear dynamics, relative impermeability of the corneal epithelial membrane and the high efficiency of the blood–ocular barrier. The majority of ophthalmic dosage forms administrated in the form of highly concentrated eye drops suffer from short precorneal residence time of eye drops which is associated with low corneal drug absorption and also ocular and systemic side effects. Furethermore, in order to achieve therapeutic effect, frequent administration of these concentrated solutions is required which results in short residence of high drug concentration in the tear film followed by long periods of underdosing (1,2). Therefore, in addition to the conventional ophthalmic dosage forms such as solutions, gels, ointments and aqueous suspensions, numerous novel ocular drug delivery systems have been developed to achieve higher bioavailability, controlled ocular delivery, patient compliance and less side effects that in situ gelling polymers, micro/nanoparticles, micro/nanoemulsions, nanosuspensions, liposomes, dendrimers and niosomes, and ocular films are amongst them (3-8). Ocular films, solid devices placed in the cul-de-sac of the eye, are more advantageous due to the more contact and prolonged retention of devices and a controlled release, the ensured effective drug concentration in the eye, more accurate pharmaceutical dosing and less systemic side-effects. Furthermore, solid devices offer more shelf life and give the advantage of biodegradability or solubility and there is no need to remove them from the eye. Nevertheless, despite all mentioned advantages, ocular films have not been widely used so far in ocular therapy (9, 10).

Diclofenac sodium, a nonsteroidal anti-inflammatory drug which is a derivative of phenyl acetic acid, is applied topically as a 1 mg/mL aqueous solution in the eye to manage pain in corneal epithelial defects in surgery or accidental trauma and symptomatic relief of seasonal allergic conjunctivitis. It can also be used to prevent intra-operative miosis during cataract surgery, and also to treat postoperative chronic inflammation and non-infectious ocular inflammation (11, 12). 

As mentioned, most of the dose applied topically in the eye from such solutions is lost due to pre-corneal losses. In the literature, there are studies done by pharmaceutical scientists on diclofenac such as liposomes (6), nanosuspensions (8) and polymeric nanoparticles (3, 5). However, the limitation of the short residence time of colloidal systems in the ocular mucosa still exists. Ideal ocular delivery systems are easy to administer, require decreased administration frequency and provide controlled and possibly sustained drug release to increase therapeutic efficacy and patient compliance (13).

The present study was aimed to prepare diclofenac sodium ocular films with the target of increasing the contact time and offering a controlled release pattern which further could improve patient compliance, reduce the frequency of administration, and obtain greater therapeutic efficacy. Therefore, diclofenac sodium ocular film utilizing various HPMC and Eudargit polymers was prepared and physicochemical parameters such as film thickness, uniformity of weight, drug content, swelling index, mucoadhesion time and *in-vitro* release study were evaluated.

## Experimental

*Materials*

Diclofenac Sodium was obtained from Daroupakhsh co., Iran. Hydroxypropylmethylcellulose (HPMC) 4000 cps was purchased from Seppic, France. HPMC 15000 cps and HPMC 100000 cps were purchased from Shandong Co., China. Eudragit RL PO and Eudragit RS PO were obtained from Rohm Pharma, Germany. Polyvinyl pyrolidone K30 (PVP K30) and sodium carboxy methylcellulose (Na CMC) were obtained from Blanver Co., Brazil. Polyethylene glycol 400 (PEG 400), Glycerin and Triethyl citrate (TEC) and all other reagents and solvents of analytical grade were purchased from Merck Co., Germany.

*Ocular diclofenac sodium film preparation*

Diclofenac sodium ocular films were prepared by solvent casting evaporation technique. The composition of prepared polymeric ocular films has been summarized in Table 1. Differnet polymers including HPMC 4000 cps (HPMC 4K), HPMC 15000 cps (HPMC 15K), HPMC 100000 cps (HPMC 100K), Na CMC, PVP 30K, EU RS PO and EU RL PO were utilized as film forming polymers. The polymer solution in distilled water, ethanol or hydroalcoholic solvent at room temperature was prepared using a magnetic stirrer and 30% w/w plasticizer of dry polymer (glycerin, TEC and PEG 400) was added to the polymer solution under stirring condition to produce flexible films as well as to protect the polymeric inserts from being brittle upon storage. The weighed amount of diclofenac sodium was added to above solution and stirred for 6 h to obtain uniform dispersion. After proper mixing, the casting solution was poured into a clean glass Petri dish (area 15.9 cm^2^) and covered with an inverted funnel to allow slow and uniform evaporation at room temperature for 48h. The dried films were cut into pieces of definite size (1 cm^2^) containing 600 µg/1cm^2^. 

**Table 1:** Composition of Diclofenac sodium ocular films

Formulation Code	Drug (mg)	HPMC 4k (mg)	HPMC 15K (mg)	HPMC 100K (mg)	Na CMC (mg)	PVP 30K (mg)	EuRL PO (mg)	EuRS PO (mg)	Plasticizer 30% W/W	Drug:polymer ratio
A1	9.6	180	-	-	-	60	-	-	G	1: 25
A2	9.6	198	-	-	-	66	-	-	G	1: 27.5
A3	9.6	216	-	-	-	72	-	-	G	1:30
B1a	9.6	432	-	-	-	-	-	-	G	1:45
B1b	9.6	-	432	-	-	-	-	-	G	1:45
B1c	9.6	-	-	432	-	-	-	-	G	1:45
B2a	9.6	216	-	216	-	-	-	-	G	1:45
B2b	9.6	-	-	192	192	-	-	-	G	1:40
B2c	9.6	-	192	-	192	-	-	-	G	1:40
C1a	9.6	180	-	-	-	-	60	-	G	1:25
C1b	9.6	216	-	-	-	-	72	-	G	1:30
C1c	9.6	252	-	-	-	-	84	-	G	1:35
C1d	9.6	270	-	-	-	-	90	-	G	1:37.5
C2a	9.6	144	-	-	-	-	144	-	G	1:30
C2b	9.6	168	-	-	-	-	168	-	G	1:35
C2c	9.6	180	-	-	-	-	180	-	G	1:37.5
C3a	9.6	72	-	-	-	-	216	-	G	1:30
C3b	9.6	84	-	-	-	-	252	-	G	1:35
C3c	9.6	84	-	-	-	-	252	-	PEG 400	1:35
D1a	9.6	180	-	-	-	-	-	60	G	1:25
D1b	9.6	216	-	-	-	-	-	72	G	1:30
D1c	9.6	252	-	-	-	-	-	84	G	1:35
D2a	9.6	168	-	-	-	-	-	168	G	1:35
D2b	9.6	192	-	-	-	-	-	192	G	1:40
D2c	9.6	216	-	-	-	-	-	216	G	1:45
D3a	9.6	84	-	-	-	-	-	252	G	1:35
D3b	9.6	84	-	-	-	-	-	252	TEC	1:35
D3c	9.6	84	-	-	-	-	-	252	G:TEC (1:1)	1:35
D3d	9.6	84	-	-	-	-	-	252	PEG	1:35

*Characterization of prepared ocular films*

*Physical characterization*

The physical characteristics of ocular films such as color, texture, flexibility and appearance were evaluated. 

*Uniformity of weight*

From each batch, 3 films were weighed individually using digital balance (Sartorius, Germany). The mean weight of the films was recorded. 

*Uniformity of thickness*

The thickness of films was determined using a Vernier caliper (Mitotoyo, Japan). For each formulation, the thickness of 3 randomly selected films was tested (9).

*Drug content detemination*

Ocular films (3 samples) were taken from each batch and dissolved using 50 mL of isotonic phosphate buffer pH 7.4 (tear fluid) into volumetric flask. The absorbance of solution after filteration and required dilution was measured by UV-VIS spectrophotometer (Shimadzu, Japan) at 283 nm. The mean drug content of films was determined considering the concentartion of the solution and the number of films dissolved.

*Swelling index test*

In order to measure the bulk hydrophilicity and hydration of films, swelling test was done as drug release from the polymeric matrix is affected by swelling. To test the swelling of diclofenac sodium films, three films of each formulation were weighed and put in a mesh basket and inserted into phosphate buffer saline (PBS) of pH 7.4 maintained at temperature of 32±0.5 °C. At time intervals of up to 90 min, the films were removed, wiped with lint-free tissue to remove excess surface PBS, weighed, and then returned back to the same container (13). 

The swelling index was calculated using the following equation based on degree of fluid uptake: 

$\mathrm{Swelling index}=\frac{W_{t}-W_{0}}{W_{0}}\times 100$

Where *W*_0_ is the initial weight of the sample and *W*_t_ is its weight at time t. 

*In-vitro drug release study*

The *in-vitro* drug release from different ocular films was studied using the vial method. Each film was placed in a vial containing 10 mL of simulated tear fluid (pH 7.4) which was previously warmed at 37 ± 1°C. These vials were positioned over a *Kottermann 4020 shaker*. To simulate the eye blinking, the shaker was kept at its minimum shaking speed. Aliquot of samples at specific time intervals was withdrawn and the equivalent amount of fresh fluid was replaced. The samples were analyzed at 283 nm using UV Spectrophotometer after appropriate dilutions against reference using isotonic phosphate buffer pH 7.4 as blank.

*Drug release kinetics*

The *in-vitro* drug release results were fitted with different kinetic models such as zero order (% release vs. t), first order (log % release vs. t), Higuchi matrix (% release vs. t^0.5^) to understand the kinetics and mechanism of drug release. Data of drug release was further analyzed by Peppas equation, M_t_/M_∞_ = kt^n^, where M_t_ is the released drug amount at time t and M_∞_ is the released amount at time ∞, the M_t_/M_∞_ is the released drug fraction at time t, k is the kinetic constant and n is the diffusional exponent, a measure of the primary mechanism of drug release. The plots of above models were analysed by regression analysis and the regression coefficient (*r*^2^) values were calculated for the linear curves obtained (9, 13). 

*Mucoadhesion study*

The mucoadhesion time was studied (in triplicate) by application of ocular films on a freshly cut sheep eyelid. Ocular film was attached to the mucosal surface of the eyelid fixed on the bottom of a beaker by applying a light force with a fingertip for 20 s. The beaker was filled with 100 mL of bicarbonate Ringer solution pH 7.4 and stirred at a rate of 150 rpm at room temperature (14, 15). Mucoadhesion time was the time needed for complete detachment of the film from the mucosal surface.

## Results and Discussion

*Physical characterization*

According to the literature, the success of film formation is proved by the fact that the prepared films are smooth in texture, translucent and uniform without any visible cracks or imperfections (16, 17). Regarding this, all prepared films were visually inspected. With exception of group A formulations (containing less than 1:10 drug: polymer) which were very soft and sticky, the rest of the films were homogeneous, translucent and flexible. Futhermore, their homogeneous and continuous surface without any crack or phase separation between the matrix and drug was achieved. This indicates the uniform distribution of the drug and polymers.

*Uniformity of weight*

The weight of ocular films in each batch was found to be uniform and in the range (Table 2). The weight uniformity of the films indicates the good distribution of the polymer, drug and plasticizer.

*Uniformity of thickness*

The thickness of ocular films in each batch varied in the range as expected (Table 2). The formulations had low standard deviation values which indicated the uniformity of the films.

*Drug content determination*

The drug content of ocular films has been presented in Table 2. As evident, the drug content varied from 96.9 ± 0.96 % to 102.10 ± 0.76% and was consistent in different formulations which indicated the fact that the drug was uniformly distributed in the polymeric matrix and the preparation method gave reproducible results.

*Swelling index*

As can be seen in Table 2 and as expected, formulations with HPMC had more swelling index. In group B formulations, cellulose derivatives, especially Na CMC had great effect on swellability (11 times increase in film weight), and Eudragits resulted in less swelling index. By applying Eudragit RS PO, it is even more decrease due to difference in chemical structure of applied Eudragits. The least amount of swelling index is achieved in group D (4 times weight increase in film). Increasing Drug: polymer ratio resulted in less swellability which can be justified by thickness increase and less water accessibility. 

*Mucoadhesion study*

The mucoadhesion time of ocular films (in triplicate) on a freshly cut sheep eyelid was studied and it was proved that in all formulations with glycerin as plasticizer, mucoadhesion time was 10 hours. However, formulations with PEG 400 had no mucoadhesion. Meanwhile, utilizing TEC single resulted in a 3-5 hour mucoadhesion and when it was combined with glycerin (1:1), the time reached to 7 hours.

**Table 2:** Physicochemical characterization of Diclofenac Na ocular films.

Formulation	Weight (mg)	Thickness (µm)	Drug content (%)	Swelling index (%)
A1	10.33 ± 0.76	75 ± 5	100.63 ± 0.65	705 ± 11.4
A2	11.60 ± 0.37	85 ± 5	98.5 ± 0.54	598 ± 6.1
A3	12.36 ± 0.55	93 ± 5.77	101. 90 ± 0.80	412 ± 5.67
B1a	20.96 ± 0.15	148. 33 ± 2.88	99.00 ± 0.59	750 ± 8.9
B1b	21.36 ± 1.48	175.00 ± 8.02	98.75 ± 0.67	1110 ± 13.4
B1c	11.93 ± 0.40	126. 00 ± 3.73	100.06 ± 0.70	1117 ±14.2
B2a	18.93 ± 1.33	133.00 ± 5.77	98.12 ± 0.80	610 ± 7.9
B2b	14.26 ± 1.10	116 ± 4.32	99.96 ± 0.49	1580 ± 16.8
C1a	13.55 ± 0.25	100.00 ± 5.5	96.9 ± 0.96	799 ± 6.8
C1b	14.23 ± 0.02	105.00 ± 8.66	98.63 ± 0.84	761 ± 5.9
C1c	14.5 ± 1.85	113.00 ± 10.40	98.97 ± 0.99	520 ± 4.6
C1d	16.76 ± 1.26	118.00 ± 7.63	102.10 ± 0.76	601 ± 5.1
C2a	11.46 ± 1.10	95.50 ± 5.00	98.55 ± 0.27	608 ± 6.2
C2b	16.52 ± 0.56	120.00 ± 0.01	99.99 ± 0.76	592 ± 6.9
C2c	13.83 ± 0.20	113.30 ± 2.80	99.97 ± 0.89	589 ± 4.9
C3a	10.20 ± 0.41	88.33 ± 5.77	98.43 ± 0.07	445 ± 5.5
C3b	14.76 ± 0.65	115.00 ± 5.00	97.56 ± 0.55	454 ± 4.8
D1a	10.00 ± 0.95	85.00 ± 10.00	98.50 ± 0.11	690 ± 7.9
D1b	11.20 ± 1.05	91.66 ± 2.88	98.86 ± 0.49	601 ± 6.8
D1c	12.46 ± 1.07	98.33 ± 7.63	99.80 ± 0.98	580 ± 5.8
D2a	17.20 ± 0.84	115.00 ± 5.00	102.05 ± 0.87	501 ± 4.9
D2b	17.76 ± 0.59	141.00 ± 4.18	99.12 ± 0.15	578 ± 5.1
D2c	18.66 ± 1.15	150.00 ± 0.00	98.98 ± 0.23	703 ± 5.6
D3a	13.33 ± 0.61	160.00 ± 8.66	98.95 ± 0.89	402 ± 3.7
D3b	14.16 ± 1.30	160.00± 10.00	99.43 ± 0.64	410 ± 3.4
D3c	17.43 ± 1.75	171.66 ± 10.27	97.97 ± 0.22	421 ± 3.6
D3d	13.95 ± 0.87	165.00 ± 5.77	97.99 ± 0.46	427 ± 4.1

Values as Mean ± SD (n=3)

*In-vitro drug release studies*

In polymeric matrices, drug release is elicited by water accessibility into the matrix, breaking the polymer–polymer bonds and simultaneously leading to the formation of water–polymer bonds, which separates polymer chains, and swells to form a gel. The drug diffuses from gel network to the medium with a diffusion rate which is dependent on its diffusion ability through the gel and its concentration gradient. Concurrently, the rate of gel matrix erosion depends on medium hydrodynamics and molecular weight of polymer. Therefore, the drug release profile mainly depends on relative rates of these processes (13, 14, and 17).

In this study, all of the successfull ocular film formulations, with proper texture and thickness were subjected to *in-vitro* drug release studies (A1-D3). In Table 3, cumulative drug release percent of diclofenac sodium in groups A and B have been summarized. As it has been depicted, group A formulations which were prepared by utilizing HPMC 4000 cps and PVP 30K released more than 50% of the drug from their matrices within 1 hour and the remaining drug was delivered in less than 4 h of the experiment. It is quite evident that both HPMC and PVP 30K were not able to effectively modulate diclofenac sodium release. In group B formulations, HPMC polymers and Na CMC, as single or combination polymers were applied and as can be seen in Table 3, these formulations were not efficient enough to contorl the drug release. This can be explained by rapid water uptake which is followed by the rapid erosion and dissolution of hydrated matrices due to the high solubility of the polymer which caused relaxation and disentanglement of polymer chains and the formation of loose network, therefore diclofenac rapidly diffused to the release medium. On the other hand, the release from HPMC 100K formulations was fast at first due to the late hydration of heavy chains of high viscosity grade of HPMC. It resulted in more freedom of drug molecules to diffuse from the outer surface/layers of the film. The release was incomplete later due to the more chain entanglement and a thicker gel formation after hydration and drug molecules entrapped in gel network and lost the ability to diffuse. Furthermore, Na CMC entraps the drug more, decreases the drug diffusivity and finally releases the drug less due to its ionic structure and ionic interction with diclofenac ions. 

**Table 3:** Cumulative drug release percent of Diclofenac Na from ocular films.

Formulation	% CR at different time intervals (h)						
	0	0.5	1	2	3	4	5
A1	0	40.63± 2.55	78.44 ± 4.58	98.93 ± 1.66	-	-	-
A2	0	71.28± 5.11	90.38 ± 9.49	106.2 ± 5.79	-	-	-
A3	0	37.08± 3.75	55.73 ± 4.40	78.80 ± 4.67	92.38± 9.18	99.70 ± 1.87	-
B1a	0	40.63± 2.55	78.44 ± 4.58	98.93 ± 1.66	100.02±4.30	102.66 ±1.93	-
B1b	0	34.67± 4.39	56.44 ± 8.37	86.81 ± 4.01	102.86±2.30	103.04±1.79	-
B1c	0	30.99± 0.55	53.46±4.23	68.07±2.67	75.63 ± 3.03	79.67 ± 2.67	-
B2a	0	41.21± 2.13	77.43 ± 4.58	97.97 ± 1.64	101.23±3.40	101.41±3.21	-
B2b	0	36.95± 3.26	47.80 ±0.00	58.19 ± 0.32	61.43 ± 0.64	62.70 ± 1.47	63.13±2.44

Values as Mean ± SD (n=3)

**Figure 1:**
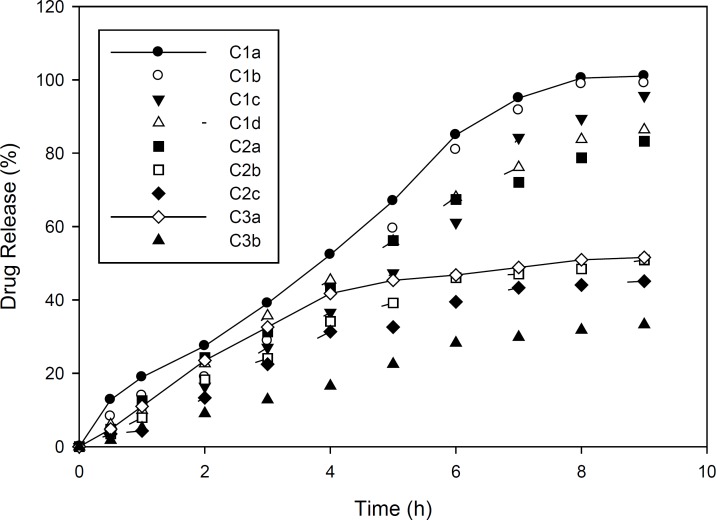
Diclofenac sodium release pattern from ocular films of formulation C in simulated tear fluid (pH 7.4) at 37±1 ^°^C (n=3, mean ± SD).

**Figure 2:**
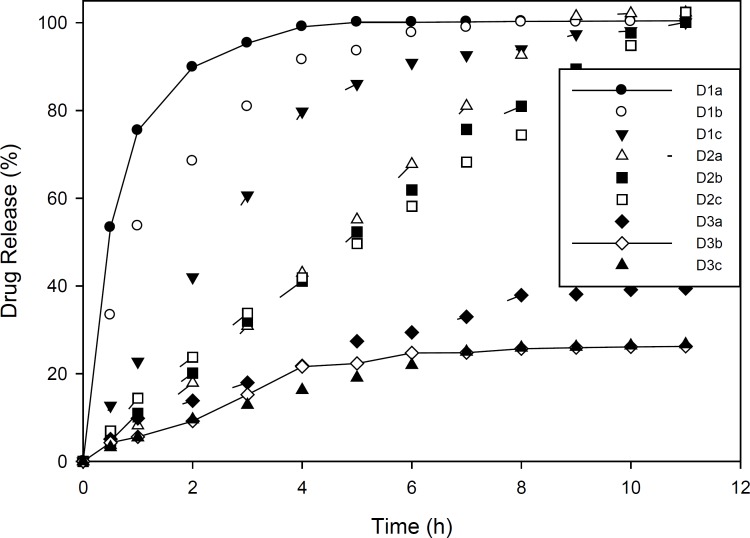
Diclofenac sodium release pattern from ocular films of formulation D in simulated tear fluid (pH 7.4) at 37±1 ^°^C (n=3, mean ± SD).

Drug release studies in Group C and D formulations are presented in Figures 1 and 2. As mentioned, group C formulations were prepared by utilizing HPMC 4K and RL PO, with different ratios (HPMC : EU RL PO, 3:1, 1:1 and 1:3) categorizing in 3 different subgroups (C1, C2 and C3). As shown in Figure 1, in these formulations, increasing drug to polymer ratio resulted in slower drug release. Furthermore, drug release rate was decreased by increasing EU RL PO, as in 1:3 ratio, t_50%_ has been achieved in 4 hours and in ratio 1:1, t_50% _has increased to 5 hours and in ratio 3:1, this time has extended to 9 hours. Finally, the total amount of released drug was not more than 34% and even did not reach 50%. This can be explained by the hydrophobic nature of Eudragit comparing HPMC and is proved by swelling studies, entrapment of drug molecules in polymeric network and less accesibilty to water channels which lead to slower release rate. In Group D formulations (Figure 2), almost the same manner of drug release can be seen. However, in 1:3 Eudragit RS PO: HPMC ratio comparing Eudragit RL PO, Eudragit RS PO resulted in faster drug release due to less quarterny amonium groups in its chemical structure and in polymer network as well as less ionic interaction with drug molecules. In 3:1 Eudragit RS PO: HPMC ratio, the behaviour is vice versa, as it lead to slower and incomplete release, *i.e*, releasing 25% in 7 hours and not more because of less wetability when it is the majority of polymer matrix (18-20).

*Plasticizer influence*

In order to study the plasticizer influence on physicochemical characteristics and drug release, Glycerin, PEG 400 and TEC were utilized as single or combination plasticizers with equal ratios in group D formulations. The ocular films prepared by TEC had more clarity in comparison with Glycerin. However, TEC films lost their flexibility in dissolution medium and it had negative effect on drug release, which can be due to the insolubility of TEC comparing Glycerin solubility in water. Formulations diclofenac PEG400 had more clarity in comparison with Glycerin, but showed much less mucoadhesion property. Therefore, glycerin was chosen as the plasticizer in this study.

*Drug release kinetics*

**Figure 3:**
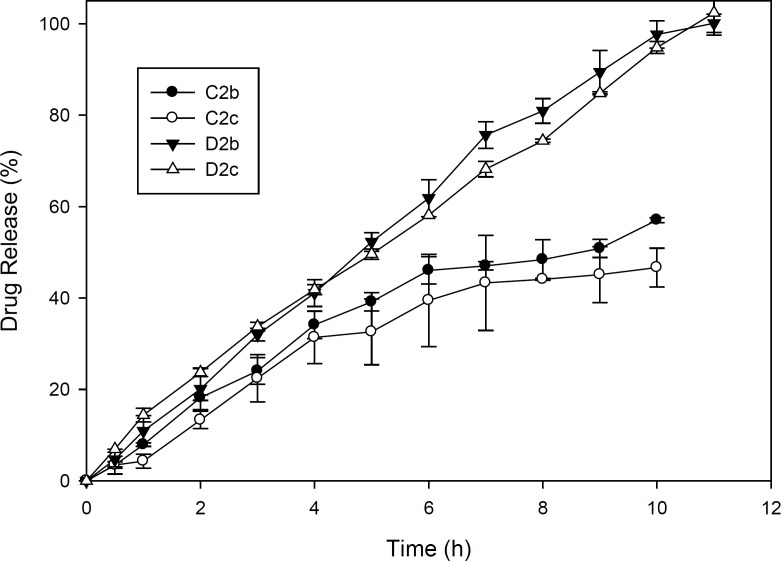
Diclofenac sodium release pattern from ocular films of formulation C2 and D2 in simulated tear fluid (pH 7.4) at 37±1 ^°^C (n=3, mean ± SD).

In order to investigate drug release kinetics, the release constants were calculated from the slope of the respective plots and the results of formulations D2b and D2c were summarized in Table 4 because these formulations were superior to others considering the physicochemical characteristics and release behaviour as can be seen in Figure 3. In these 2 ocular film formulations perfoming regression analysis, higher correlation was observed with respect to zero order plots (*r*^2^ > 0.99). It was confirmed by zero order plots that the drug diffused slowly from ocular fims. In planar geometry by applying Korsmeyer-Peppas model, the value of n = 0.5 implies a Fickian diffusion, 0.5 < n < 1.0 indicates anomalous (non-Fickian) transport, and n = 1 indicates case II (relaxation controlled) transport. In the present films, this value was found in the range of 0.8473–1.0092 which implied that the release mechanisms followed anomalous (non-Fickian) transport and zero order release (case II transport) (9, 21).

**Table 4:** Kinetic data of drug release from D2b and D2c formulations

F	Zero order	First order	Higuchi	Hixon-crowell	Korsmeyer-peppas model		
R^2^					R^2^	n	Order of release
D2b	0.9960	0.8450	0.9780	0.9720	0.9975	1.0092	Super case- II transport
D2c	0.9970	0.8620	0.9750	0.9500	0.9978	0.8473	non-Fickian diffusion

## Conclusions

Various batches of diclofenac sodium ocular films applying hydrophylic HPMC and Eudragits were prepared using solvent casting method and then were evaluated.

D2b and D2c formulations containing Eudragit RS PO and HPMC with ratio 1:1 and glycerin as plasticizer satisfied all pharmaceutical parameters of ocular films and demonstrated the controlled release of the drug *in-vitro* over the period of 11 hours with zero order kinetics. 

The results of the present study revealed that polymer types as well as their properties, drug: polymer ratio and plasticizer type to formulate the ocular films are important criteria which influence the film swelling and thus affect the drug release. Our study suggested that the mucoadhesive feature and the sustaining effect on drug release obtained by proper optimization to prepare suitable film can be exploited as a potential candidate to formulate sustained release diclofenac ocular films.
